# Detection and Identification of *Paragyliauchen* sp. (Trematoda) and Bacteria in Diseased *Centropyge bicolor* from the European Marine Aquarium Trade

**DOI:** 10.3390/pathogens15030245

**Published:** 2026-02-25

**Authors:** Leszek Guz, Anna Pastuszka, Michał Torbicz, Michał Oszust

**Affiliations:** 1 Department of Biology and Fish Diseases, Faculty of Veterinary Medicine, University of Life Sciences, Akademicka 12, 20-950 Lublin, Poland; leszek.guz@up.lublin.pl; 2 Student Scientific Association of Veterinary Biology, Faculty of Veterinary Medicine, University of Life Sciences, Akademicka 12, 20-950 Lublin, Poland; michaltorbicz12@gmail.com (M.T.); oszust.michal@gmail.com (M.O.)

**Keywords:** fish, fluke, co-infection, *Aeromonas*, *Shewanella*, *Brevundimonas*

## Abstract

During the examination of a bicolor angelfish (*Centropyge bicolor*) imported from the Philippines and intended for sale on the Polish market, clinical signs of weakness and respiratory distress were observed. Mild hyperemia was noted along the lateral sides of the body and around the mouth. Necropsy revealed the presence of five orange-colored trematodes in the intestinal lumen, with an average body length of 3.12 mm. Based on morphological features and molecular analyses, the parasite was identified as *Paragyiauchen* sp. The following gene loci were amplified: the gene-encoding component of the large ribosomal subunit (*28S rRNA*), the gene-encoding NADH dehydrogenase subunit 1 (*ND1*), the gene cluster *5.8S rRNA-ITS2-28S rRNA* and the gene cluster *ITS2-28S rRNA*. Bacterial species identification using MALDI-TOF MS revealed the presence of three species: *Shewanella putrefaciens* and *Brevundimonas diminuta* isolated from the head kidney, and *Aeromonas caviae* isolated from the liver. This study documents the first detection of representatives of *Paragyliauchen* genus in *C. bicolor* imported to Europe and highlights the potential risk of introducing new parasites and opportunistic bacterial pathogens through the ornamental fish trade. These findings emphasize the need for parasitological and microbiological screening of imported ornamental fish.

## 1. Introduction

Gyliauchenidae Fukui, 1959 (Syns.: Dissotrematidae Goto & Matsudaira, 1918; Robphildollfusiidae Paggi & Orecchia, 1963) is a family of trematodes that mainly parasitize herbivorous fish species endemic to coral reef environments in the Indo-West Pacific region [1]. These flukes inhabit the gastrointestinal tract of fish belonging to families such as Acanthuridae, Chaetodontidae, Pomacanthidae, Scaridae, Siganidae, and Zanclidae, although small numbers of individuals have also been recorded in hosts from the Labridae and Pomacentridae families [1]. The Gyliauchenidae family has a complex and still not fully understood taxonomic structure [2]. Initially, five subfamilies were proposed within Gyliauchenidae: Petalocotylinae Ozaki, 1934; Robphilidollfusiinae Paggi & Orecchia, 1963; Apharyngogyliaucheninae Yamaguti, 1942; Ichthyotreminae Caballero & Bravo-Hollis, 1952; and Gyliaucheninae Fukui, 1929. Karar et al. [2] treat the subfamilies Apharyngogyliaucheninae and Robphilidollfusiinae as junior synonyms of Gyliaucheninae. These authors also propose the establishment of a distinct subfamily, Paragyliaucheninae, which includes only the genus *Paragyliauchen* Yamaguti, 1934 [2]. Thus, they recognize only four subfamilies within Gyliauchenidae: Gyliaucheninae, Petalocotylinae, Robphilidollfusiinae, and Paragyliaucheninae. The genus *Paragyliauchen* is distinguished primarily by the position of the ventral sucker located in the anterior third of the body and the distribution of vitelline glands from the esophageal bulb to the level of the gonads [2,3]. A detailed diagnosis of the subfamily Paragyliaucheninae is provided by Karar et al. [2].

According to WoRMS, the genus *Paragyliauchen* comprises two species with valid taxonomic status—*Paragyliauchen arusettae* Machida, 1984, and *Paragyliauchen chaetodonis* Yamaguti, 1934—whereas Hall [1] provided evidence suggesting a higher species diversity within the genus.

Flukes belonging to the genus *Paragyliauchen* are pyriform, with the ventral sucker clearly separated from the posterior end of the body. The esophagus is slightly S-shaped, and an esophageal bulb is present. The ceca terminate at the level of the ventral sucker, and the testes are symmetrical, located posterior to the ventral sucker. In *P. chaetodontis*, the vitellaria are follicular, and the genital pore is located anterior to the cecal bifurcation, whereas in *P. arusettae*, the vitellaria are ramiform and the genital pore is posterior to the cecal bifurcation [4].

The life cycle of members of the family Gyliauchenidae is not fully understood. They are hermaphroditic, and herbivorous fishes serve as the definitive hosts. In the gastrointestinal tract of the fish, the flukes produce eggs, which are excreted with the feces into the aquatic environment. Snails are presumed to act as intermediate hosts. Cercariae encyst on aquatic vegetation, forming metacercariae [5], and fish become infected through ingestion of these metacercariae. In the fish intestine, the parasites feed primarily on partially digested plant matter rather than host tissue. Typically, a few to several dozen individuals are observed in the gastrointestinal tract, especially in siganids, and they do not cause visible clinical signs [5,6].

The natural biogeographic range of *Centropyge bicolor* covers a vast area of the tropical Indo-West Pacific region, from East Africa in the west, through the north up to the Ryukyu Archipelago, and in the east to some Pacific islands [7].

Coral reef fish represent a significant component of the global aquarium trade, which involves the annual transport of tens of millions of individuals from tropical regions to consumers around the world. One of the most popular species among marine aquarium hobbyists is C. bicolor, a member of the family Pomacanthidae. This species, commonly known as the bicolor angelfish, is characterized by its distinctive, contrasting body coloration and small size, making it an attractive choice for home reef tanks. Between 2014 and 2021, a total of 1,677,523 individuals of this species were imported into Europe, with an average of 209,690 per year [8].

An increasing number of scientific reports document the identification of previously unknown fish pathogens in Europe [9,10,11,12,13], including in Poland [14,15,16,17], highlighting the need for comprehensive monitoring of the health status of imported fish. As part of this monitoring, it is also important to examine fish for the presence or potential occurrence of pathogenic bacteria, which may pose a threat both to fish health and to local aquatic ecosystems, as well as to ornamental fish farming.

To date, there are no published reports confirming the presence of *Paragyliauchen* species in marine ornamental fish in Europe. This study aimed to identify and characterize the trematodes and bacteria present in a fish imported from the Philippines and to document their occurrence and distribution within the host.

## 2. Case Description and Methods

A fish of the species *C. bicolor* (common name: bicolor angelfish) (n = 1), belonging to the family Pomacanthidae, was submitted to the Department of Biology and Fish Diseases, Faculty of Veterinary Medicine, University of Life Sciences, in Lublin, Poland, for diagnostic necropsy. The examined fish originated from an import shipment from the Philippines by a local marine fish distributor. The disturbing symptoms that the fish showed and which prompted a post-mortem examination included weakness, lack of appetite, respiratory distress, and mild skin hyperemia, especially around the head and gill cover.

All procedures were conducted in accordance with Directive 2010/63/EU on the protection of animals used for scientific purposes and in line with current recommendations concerning fish welfare in experimental research [18,19,20]. The diseased fish was euthanized in accordance with the American Veterinary Medical Association guidelines [21].

A diagnostic necropsy was then performed, including an assessment of external morphological features (skin color, presence of skin lesions, condition of fins, eyes, and gills) and examination of internal organs within the body cavity (liver, kidneys, spleen, intestines, swim bladder, reproductive organs, and heart).

During the necropsy, skin scrapings, gills, and intestinal contents were also collected for parasitological examination. All procedures were conducted under aseptic conditions and in compliance with biosecurity protocols.

Additionally, to identify potential bacterial etiological agents, sterile samples were taken from the liver and kidney and cultured on tryptic soy agar (TSA). The plates were incubated at 28 °C for 48 h. Colony growth was evaluated macroscopically, and isolates were preserved for further identification. The resulting colonies were identified using matrix-assisted laser desorption/ionization–time-of-flight mass spectrometry (MALDI-TOF MS) (Bruker Daltonik GmbH, Bremen, Germany), as described by Pastuszka et al. [22] and Guz et al. [17,23]. Bacterial mass spectra were analyzed using MALDI Biotyper 3.1. The interpretative score criteria recommended by the manufacturer were applied as follows: a score ≥ 2.300 indicates highly probable species-level identification; a score of 2.000–2.299 indicates secure genus-level and probable species-level identification; a score of 1.700–1.999 indicates probable genus-level identification; and a score < 1.700 indicates unreliable identification.

In the case of bacterial isolates for which identification based on the ribosomal protein profile was not sufficient, genomic DNA isolation and amplification of the *16S rRNA* gene fragment were performed using conventional PCR. In this method, a pair of standard primers 27F (5ʹ AGA GTT TGA TCM TGG CTC AG 3ʹ) and 1429R (5ʹ TAC GGY TAC CTT GTT ACG AC TT 3ʹ) were used, and the composition of the reaction mixture and the amplification protocol were as previously described [24]. The obtained amplicons (1500 bp) were eluted from the agarose gel and then sequenced using the Sanger method (Genomed, Warsaw, Poland), performed with a forward and reverse primer for PCR. The sequences prepared were compared for final reference with the sequences deposited in GenBank.

Additionally, the biochemical profiles of the identified bacterial isolates were examined using commercial API 20E tests (Biomerieux, Craponne, France). The procedure was performed according to the manufacturer’s guidelines, with *Escherichia coli* ATCC 25,922 serving as the reference strain.

Based on the genetic method, the species affiliation of flukes isolated from the digestive tract of the fish *C. bicolor* was also confirmed. Isolation of genetic material was performed similarly to bacterial isolates using the commercial Genomic Mini Kit (A&A Biotechnology, Gdansk, Poland), according to the protocol attached by the manufacturer. The obtained DNA was eluted from the minicolumn resin using a Tris buffer solution heated to 70 °C (100 µL), and then the conventional PCR reaction and semi-nested PCR reaction were started. The following gene loci were amplified: the gene-encoding component of the large ribosomal subunit (*28S rRNA*), the gene-encoding NADH dehydrogenase subunit 1 (*ND1*), the gene cluster 5.8S rRNA-ITS2-28S rRNA and the gene cluster ITS2-28S rRNA. The sequences of primers used in the PCR reaction, reaction conditions and the sizes of the obtained amplicons (bp) are given in Table 1.

In the next step, the amplification products were purified from the agarose gel residues by Gel-Out Kit (A&A Biotechnology, Poland) and sent for Sanger sequencing (Genomed, Warszawa, Poland). Sequences developed using dedicated programs (Finch TV 1.4.1 and BioEdit) and subjected to comparative identification using the BLAST algorithm version BLAST+ 2.17.0 were deposited in GenBank as records but were also used together with their closest matches to construct phylogenetic trees using the neighbor-joining method. Sequences were previously aligned using ClustalW and the final cluster analysis by MEGA 11 with 500 bootstrap replicates and the Maximum Composite Likelihood algorithm to compute the evolutionary distances.

## 3. Results

**Figure 1:**
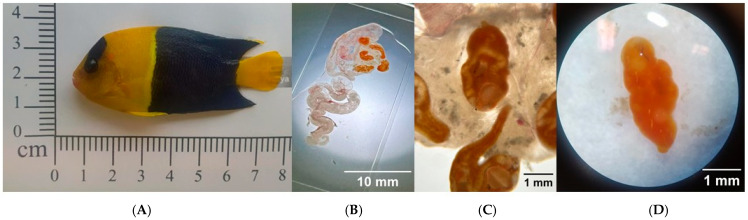
(**A**) Diseased *C. bicolor* fish with visible hyperemia on the body surface—indicated by arrows. (**B**–**D**) Orange trematodes in the digestive tract of the examined fish, visible under a stereoscopic microscope, *Paragyliauchen* sp. trematodes.

During the examination of the marine fish *C. bicolor*, imported from the Philippines and intended for sale on the Polish market, clinical signs of disease were observed. The fish exhibited marked weakness and signs of respiratory distress. Mild hyperemia was noted on the skin along the lateral sides of the body and around the mouth. The gills showed no visible macroscopic lesions. Diagnostic necropsy revealed the presence of five orange-colored trematodes in the intestinal lumen, with an average body length of approximately 3.12 mm (specifically: 2.8, 3.1, 3.1, 3.3, 3.3 mm) (Figure 1). Mild intestinal hyperemia and pinpoint hemorrhages were observed in the small intestine. No macroscopic abnormalities were noted in the remaining internal organs.

Microscopic examination of the parasites and analysis of morphological features described by Karar et al. [2], Nahhas and Wetzel [4], and Hall and Cribb [3,28] suggested that the isolated parasites were characteristically similar to members of the species *Paragyliauchen arusettae*.

To determine the taxonomic identity of the parasite isolated from the intestine of C. bicolor, phylogenetic analyses were performed based on three molecular markers: fragments of the *28S rRNA* gene, NADH dehydrogenase subunit 1 (*ND1*), and the *ITS2* region together with the *5.8S rRNA* gene. The analyzed genes (Table 1) were amplified from parasite DNA and subsequently sequenced. The tested isolate showed similarity of genetic sequences to the genus *Paragyliauchen*. BLAST analysis showed that the *28S rRNA* gene sequence is similar to that of *Paragyliauchen arusettae* (AY222235, a strain isolated from *Pomacanthus sexstriatus* in Australia), showing 98.95% identity (differing by 13 nucleotides) (Table 2). The *ND1* gene sequence was also similar to *Paragyliauchen arusettae* (FJ88788465, strain isolated from *P. sexstriatus* in Ningaloo, Australia), with 98.94% identity (compared sequences differed by five nucleotides) (Table 2).

The *ITS2 rRNA* sequence showed similarity to *Paragyliauchen atractus* (AY379507, strain isolated from *Centropyge* sp. in Lizard Island, Great Barrier Reef, Queensland, Australia), with 95.68% identity (the sequences differed from each other by 18 nucleotides) (Table 2). The 5.8S-ITS2 gene cluster also showed similarity to *Paragyliauchen atractus* (AY379507), with 94.34% identity (the difference is also 18 nucleotides) (Table 2). It turned out to be difficult to clearly determine the species affiliation of the tested isolate based on genetic sequences, because only 22 reference gene sequences for representatives of the genus *Paragyliauchen* have been deposited in the GenBank database so far (3 sequences for *Paragyliauchen aff. chaetodontis* Type A, 5 sequences for *Paragyliauchen aff. chaetodontis* Type B, 4 sequences for *Paragyliauchen arusettae*, 1 sequence for *Paragyliauchen atractus*, 5 sequences for *Paragyliauchen fractoporus* and 4 described as *Paragyliauchen* sp.). Moreover, the degrees of identity of the sequences obtained after sequencing and compared with those available in GenBank were not higher than 98.95%. Based on the currently available genetic data, we were unable to clearly determine the species of the isolated fluke; therefore, our isolate was classified as *Paragyliauchen* sp. The genetic sequences obtained in our studies were deposited in GenBank under the sequence numbers PV746782, PV752319, PV747419 and PV747185 and described *as Paragyliauchen* sp. strain PA1.

**Figure 2:**
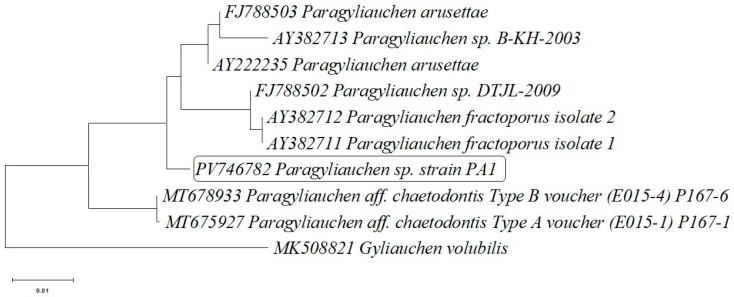
Phylogenetic tree based on the partial *28S rRNA* (large subunit ribosomal RNA) gene sequence of *Paragyliauchen* sp. strain PA1 isolated from the gut of bicolor angelfish (*Centropyge bicolor*) and the partial *28S rRNA* gene sequences of 8 other closely related *Paragyliauchen* representatives deposited in the GenBank database. The sequence obtained from *Gyliauchen volubilis* was added as an outgroup strain sequence.

The phylogenetic tree based on the *28S rRNA* gene sequences (Figure 2) showed that the analyzed isolate (PA1) clusters within a well-supported clade together with *P. arusettae* representatives previously deposited in the GenBank database (AY222235, FJ788503), *P.fractoporus* (AY382711, AY382712) and *Paragyliauchen* sp. (FJ788502, AY382713). The tree topology supports the close phylogenetic relationships with other members of the genus *Paragyliauchen* The sequence of *Gyliauchen volubilis* (MK508821) was used as an outgroup and separates from the remaining taxa, confirming its phylogenetic distinctiveness (Figure 2).

**Figure 3:**
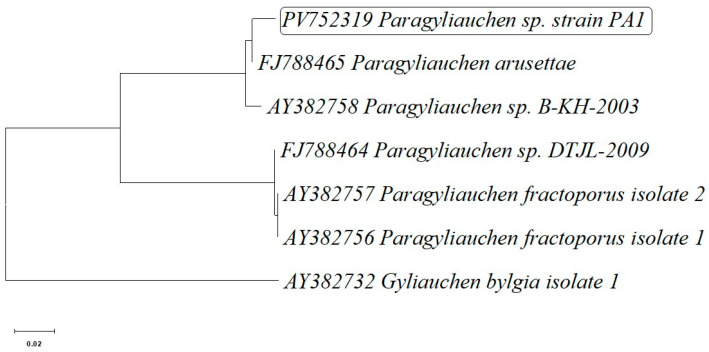
Phylogenetic tree based on the partial cds of NADH dehydrogenase subunit 1 (*ND1*) gene sequence of *Paragyliauchen* sp. strain PA1 isolated from the gut of bicolor angelfish (*Centropyge bicolor*) and partial cds of NADH dehydrogenase subunit 1 (ND1) gene sequences of 5 other closely related *Paragyliauchen* representatives deposited in the GenBank database. The sequence obtained from *Gyliauchen bylgia* was added as an outgroup strain sequence.

An additional analysis was performed based on the mitochondrial *ND1* gene sequence. The PA1 isolate (PV752319) forms a distinct and strongly supported clade with the reference sequence of *P. arusettae* (FJ788465), also showing close relationships with other representatives of the genus *Paragyliauchen*, including *P. fractoporus* and taxa identified as *Paragyliauchen* sp. DTJL-2009 and sp. B-KH-2003. The *Gyliauchen bylgia* sequence (AY382732) was used as the outgroup and displayed the greatest phylogenetic distance (Figure 3).

**Figure 4:**
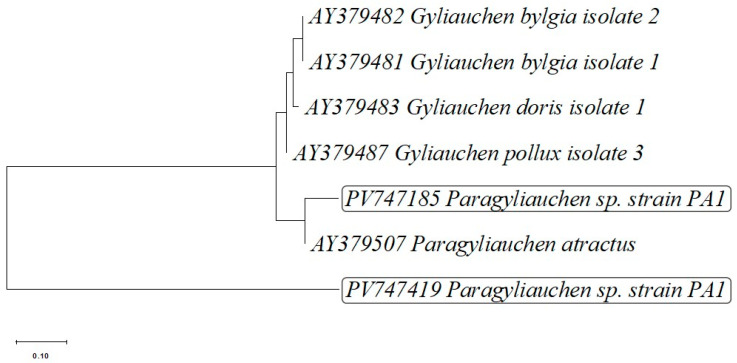
Phylogenetic tree based on the partial sequence of 5.8S ribosomal RNA gene, internal transcribed spacer 2 *(ITS2*) and large subunit ribosomal RNA gene sequence of *Paragyliauchen* sp. strain PA1 isolated from the gut of bicolor angelfish (*Centropyge bicolor*), obtained by conventional PCR, (PV747419) and partial sequence of internal transcribed spacer 2 (*ITS2*) and large subunit ribosomal RNA gene sequence, obtained by semi-nested PCR (PV747185) methods, with addition of partial 5.8S ribosomal RNA gene and internal transcribed spacer 2 (*ITS2*) gene sequences of 5 other closely related *Paragyliauchen* and *Gyliauchen* representatives deposited in the GenBank database. *Fasciola hepatica* was added as an outgroup strain sequence.

Another phylogenetic tree was constructed based on the analysis of the *ITS2* region and the *5.8S rRNA* gene (Figure 4). Sequences obtained with semi-nested PCR (PV747185) from isolate PA1 clustered together with the sequence of *P. atractus*. This is the only sequence of internal transcribed spacer 2 (*ITS2*) of members of the genus *Paragyliauchen* deposited in GenBank.

**Figure 5:**
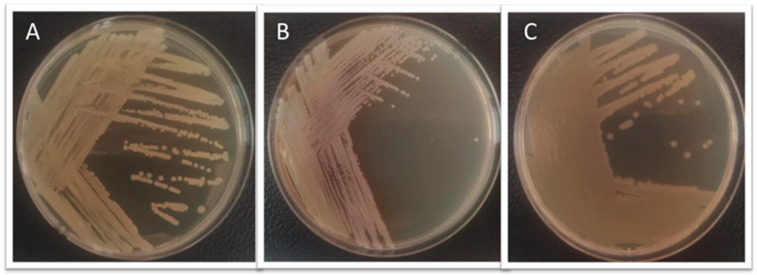
Bacteria isolated from *Centropyge bicolor* growing on TSA medium: (**A**) *Aeromonas caviae* after 24 h of growth; (**B**) *Brevundimonas dimunuta* after 48 h of growth; (**C**) *Shewanella putrefaciens* after 24 h of growth.

Swabs from the liver and the trunk kidney were aseptically collected for bacterial isolation and characterization. The samples were plated onto tryptic soy agar (TSA) and incubated at 28 °C for 24 h (Figure 5). Bacterial species identification using MALDI-TOF MS revealed the presence of three species: *Shewanella putrefaciens* and *Brevundimonas diminuta* isolated from the head kidney, and *Aeromonas caviae* isolated from the liver, with score values of 2.186, <1.699, and 2.206, respectively (Table 3).

For *B. diminuta*, the system was unable to reliably assign the isolate to a specific genus or species (score value ≤ 1.699), indicating unreliable identification (Table 3). Therefore, a fragment of the *16S rRNA* gene was amplified using conventional PCR, and the resulting amplicon was subsequently sequenced, revealing 99.55% identity of the isolate to the reference strain *B. diminuta* ATCC 11568 (Table 4).

Commercial API-20E tests were used to broadly identify the features of the bacterial isolates’ metabolism. The results are summarized in Table 5.

The isolate identified as *Aeromonas caviae* demonstrated metabolic characteristics such as the ability to produce β-galactosidase (ONPG test), the activity of arginine dihydrolase, and the ability to utilize trisodium citrate. It was positive in oxidase reaction, gelatine liquefaction tests and rapidly fermented compounds such as D-glucose, D-mannitol, D-saccharose, L-arabinose, and amygdaline. With respect to the remaining reactions in the test, the result was negative. Interpretation of the obtained profile using apiweb software API 20E version 5.0 (apiweb™ bioMerieux software, France) allowed for biochemical identification of the isolate as *Aeromonas hydrophila/caviae/sobria* (96.4% probability) (Table 5). Another isolate, genetically identified as *Brevundimonas diminuta* in the API 20E test, demonstrated only the ability to utilize trisodium citrate. This isolate was also found to be oxidase-positive. All other reactions in the test, based on the interpretation table provided by the manufacturer, were considered negative. The obtained metabolic profile did not allow for a reliable biochemical identification of the isolate. It is possible that other kits offered by bioMerieux (e.g., 20NE) would be more suitable for *Brevundimonas diminuta* (Table 5). The last isolate tested was an isolate tentatively identified as *Shewanella putrefaciens*. In this case, the interpretation of the biochemical profile based on apiweb software clearly indicated the result: *Shewanella putrefaciens* group, 97.2% probability. The isolate showed positive reactions in the oxidase test, was able to utilize trisodium citrate, and produced H_2_S.

## 4. Discussion

In the present study, we report for the first time in Europe a confirmed case of *Paragyliauchen* sp. infection in *C. bicolor*, a marine ornamental fish imported from the Philippines and intended for sale on the Polish aquarium market. The examined fish exhibited clinical signs such as weakness, respiratory distress, and mild skin hyperemia. Mild hyperemia and pinpoint hemorrhages were observed in the small intestine, while no macroscopic abnormalities were detected in the internal organs. Additionally, the presence of bacteria, including *A. caviae*, *B. diminuta*, and *S. putrefaciens*, was confirmed in the internal organs of the affected fish.

Species of the genus *Paragyliauchen* are recognized as common intestinal parasites infecting coral reef fishes, especially those belonging to the families Acanthuridae and Pomacanthidae, and are typically found in tropical Indo-Pacific regions [3,28]. The morphological characteristics of the trematodes examined in this study—including body length, coloration, and anatomical structure—corresponded to the descriptions of *P. arusettae* provided by Karar et al. [2] and Nahhas and Wetzel [4]. Molecular analysis, including sequencing of the *28S rRNA* and *ND1* gene fragments, confirmed the morphological identification, showing similarity to *P. arusettae* sequences (AY222235 and FJ788465) available in the GenBank database (Table 2). Two newly submitted sequences assigned to *Paragyliauchen* are also reported: PV747185, representing the 5.8S rRNA-ITS2-28S cluster, and PV747419, representing the ITS2-28S rRNA cluster (Table 2). The results of all three phylogenetic analyses (Figure 2, Figure 3 and Figure 4) unequivocally confirm the identification of the examined parasite as a member of the genus *Paragyliauchen*, similar to *P. arusettae*. The molecular markers used (*28S rRNA*, *ND1*) proved effective in differentiating taxa within the family Gyliauchenidae and confirmed the phylogenetic relatedness of the analyzed isolate to other representatives of the genus *Paragyliauchen*.

The observed hyperemia in the intestines of *C. bicolor* may result from the local effects of the flukes, suggesting moderate tissue irritation caused by the parasites. Although the number of parasites was relatively low, even mild intestinal infections can lead to impaired nutrient absorption and chronic weakness, particularly under stress conditions associated with transport and acclimatization. The presence of intestinal trematodes in fish is often correlated with bacterial infections in internal organs, which may be explained by several pathophysiological mechanisms, including: (1) disruption of the intestinal barrier, facilitating bacterial translocation from the gut lumen into the bloodstream and subsequently into internal organs [29]; (2) disturbance of the intestinal microbiota, leading to bacterial translocation beyond the intestine [30]; (3) modulation of the immune system, facilitating the development of bacterial infections [30]; (4) parasite-induced stress resulting in elevated cortisol levels, immunosuppression, and the emergence of secondary infections. All of these mechanisms contribute to the common occurrence of mixed infections—parasitic and bacterial—in fish disease pathology, as previously observed [17,23].

In the present study, the bacteria *A. caviae* and *S. putrefaciens* were identified, both of which have previously been reported as pathogenic agents isolated from diseased fish [31,32]. Both *A. caviae* and *S. putrefaciens* are commonly found in aquatic environments and are capable of causing opportunistic infections in fish, particularly under conditions of environmental stress or weakened immunity [33]. In contrast, *B. diminuta*, although widely distributed mainly in freshwater and natural environments [34], to the best of the authors’ knowledge, has not previously been isolated from fish. The isolation of this species in the present study may indicate its potential role as an opportunistic colonizer of damaged tissues or as a component of the environmental microbiome that acquires pathogenic significance under certain conditions. It is worth noting that *B. diminuta* has been described as an opportunistic organism in humans, especially in immunocompromised individuals [35], which may suggest a similar potential in the context of fish health.

It is important to emphasize that the isolation of the aforementioned bacteria was performed from fish showing clinical signs of disease, which simultaneously exhibited the presence of *Paragyliauchen* trematodes and bacteria in their internal organs. This coexistence of parasites and bacteria may facilitate the translocation of bacteria from the gastrointestinal tract and increase their pathogenic potential [36]. Despite the absence of visible pathological changes in the liver and kidneys, the presence of potentially pathogenic bacteria in these organs may indicate subclinical infection or an early stage of systemic infection.

## 5. Conclusions

This study documents the invasion of *Paragyliauchen*, similar to *P. arusettae*, and the bacteria *A. caviae*, *B. diminuta*, and *S. putrefaciens* in the marine fish *C. bicolor*. These infections may cause damage to the intestinal barrier, alterations in the intestinal microbiota, and immune responses, which together can facilitate the translocation of bacteria to the fish’s internal organs. Understanding these mechanisms requires further research and is crucial for assessing the impact of the studied parasites on fish health.

The analyses conducted contribute to a better understanding of health risks in marine ornamental fish introduced to trade in Europe and underscore the need for routine parasitological diagnostics during the transport and trade of tropical-origin species.

## Figures and Tables

**Table 1:** Primers used in this study to identify the gut flukes from *Centropyge bicolor*.

Target	Primer Sequence (5′-3′)	PCR Profile	Amplicon Size (bp)	Reference
ITS–2 rDNA (primers for semi-nested PCR)	F: 3S: GGT ACC GGT GGA TCA CGT GGC TAG TG GA1: AGA ACA TCG ACA TCT TGA AC (sequencing) R: ITS2.2: CCT GGT TAG TTT CTT TTC CTC CGC	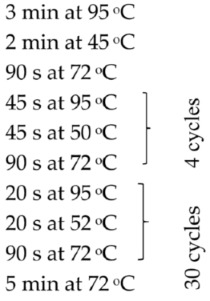	480	[25]
ITS2 (primers for conventional PCR)	F: ITS2: TGT GTC GAT GAA GAG CGC AG R: ITS2: TGG TTA GTT TCT TTT CCT CCG C	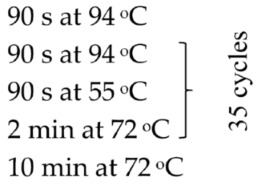	505	[26]
*28S rRNA* gene	F: ZX-1: ACC CGC TGA ATT TAA GCA TAT R: 1500R: GCT ATC CTG AGG GAA ACT ACT	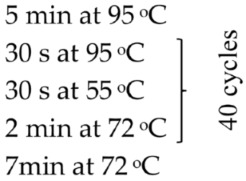	1200	[2]
*ND1* (NADH dehydrogenase subunit 1)	F: NDJ1: AGA TTC GTA AGG GGC CTA ATA R: ND1J2A: CTT CAG CCT CAG CAT AAT C	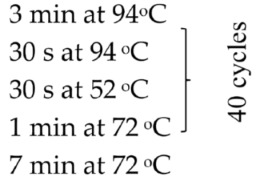	510	[27]

**Table 2:** Sequences deposited in GenBank to which partial gene sequences obtained from *Paragyliauchen* sp. strain PA1 showed the highest degree of identity.

**Accession Number: PV746782, *Paragyliauchen* sp. Strain PA1, *28S rRNA* Gene, Partial Sequence**				
Accession number of nucleotide sequence	Description	Geographical location	Host/isolation source	Percent identity (%)
AY222235	*Paragyliauchen arusettae 28S rRNA* gene, partial sequence	Ningaloo, Wesern Australia	*Pomacanthus sexstriatus*	98.95%
**Accession number: PV747419, *Paragyliauchen* sp. strain PA1, *5.8S rRNA* gene, partial sequence-*ITS2*, complete sequence-*28S rRNA* gene, partial sequence**				
Accession number of nucleotide sequence	Description	Geographical location	Host/isolation source	Percent identity (%)
AY379507	*Paragyliauchen atractus 5.8S rRNA* gene and ITS2, partial sequence	Lizard Islands, Great Barrier Reef, Queensland, Australia.	*Centropyge*	95.68%
**Accession number: PV747185, *Paragyliauchen* sp. strain PA1, ITS2-*28S rRNA* gene, partial sequence**				
Accession number of nucleotide sequence	Description	Geographical location	Host/isolation source	Percent identity (%)
AY379507	*Paragyliauchen atractus* 5.8S rRNA gene and ITS2, partial sequence	Lizard Islands, Great Barrier Reef, Queensland, Australia.	*Centropyge*	94.34%
**Accession number: PV752319, *Paragyliauchen* sp. strain PA1, NADH dehydrogenase subunit 1 (ND1) gene, partial cds, mitochondrial**				
Accession number of nucleotide sequence	Description	Geographical location	Host/isolation source	Percent identity (%)
FJ788465	*Paragyliauchen arusettae ND1* gene, partial sequence; mitochondrial	Ningaloo Reef, Western Australia	Pomacanthus sexstriatus	98.94%

**Table 3:** Identification by MALDI-TOF MS method of bacteria isolated from diseased fish *Centropyge bicolor*.

Isolate Signature	MALDI-TOF MS		Site of Infection	Host Species	Date of Sapling
	Score Values	Species ID (Based on MALDI Biotyper 3.1 Software)			
1.	2.186	*Shewanella putrefaciens* CCM 2601 CCM	Trunk kidney	Bicolor angelfish (*Centropyge bicolor*)	13-03-2025
2.	2.206	*Aeromonas caviae* CECT 838T DSM	Liver		
3.	≤1.699	Not reliable identification	Trunk kidney		

**Table 4:** Sequence deposited in GenBank to which partial *16S rRNA* gene sequence obtained from *Brevundimonas diminuta* strain BD01 showed the highest degree of identity.

Accession Number: PV812437, *Brevundimonas diminuta* BD 01, *16S rRNA* Gene, Partial Sequence				
Accession number of nucleotide sequence	Description	Geographical location	Isolation source	Percent identity (%)
NCBI Reference Sequence: NR_113238	*Brevundimonas diminuta* ATCC 11568 *16S rRNA*, partial sequence	USA	Freshwater stream	99.55%

**Table 5:** API 20E test results of bacteria isolated from *Centropyge bicolor*.

Characteristics	*Aeromonas caviae*	*Brevundimonas dimunuta*	*Shewanella putrefaciens*
Oxidase reaction	+	+	+
ONPG test (β-galactosidase)	+	-	-
Arginine dihydrolase	+	-	-
Lysine decarboxylase	-	-	-
Ornithine decarboxylase	-	-	-
Trisodium citrate utilization	+	+	+
H_2_S production	-	-	+
Urease production	-	-	-
Deamination of tryptophan	-	-	-
Indole production	-	-	-
Acetoin production (VP test)	-	-	-
Gelatin liquefaction	+	-	-
D-Glucose fermentation	+	-	-
D-Mannitol fermentation	+	-	-
Inositol fermentation	-	-	-
D-sorbitol fermentation	-	-	-
L-rhamnose fermentation	-	-	-
D-saccharose fermentation	+	-	-
D-melibiose fermentation	-	-	-
Amygdaline fermentation	+	-	-
L-arabinose fermentation	+	-	-
API-20E code number	3 206 127	0 200 004	0 600 004
API-20E identification	*Aeromonas hydrophila/caviae/sobria*	Not reliable identification	*Shewanella putrefaciens* group
API-20E % identification	96.4	-	97.2

+, indicates positives results; -, indicates negative results.

## Data Availability

The original contributions presented in this study are included in the article. Further inquiries can be directed to the corresponding author. The sequences obtained in this study were deposited in GenBank under accession numbers PV746782, PV747419, PV747185, and PV752319.

## Abbreviations

The following abbreviations are used in this manuscript:

MALDI-TOF MS	Matrix-Assisted Laser Desorption/Ionization–Time-of-Flight Mass Spectrometry
NCBI	National Center for Biotechnology Information
PCR	Polymerase Chain Reaction
BLAST	Basic Local Alignment Search Tool
IUCN	International Union for Conservation of Nature
